# Social Consequences of Ebola Containment Measures in Liberia

**DOI:** 10.1371/journal.pone.0143036

**Published:** 2015-12-09

**Authors:** Umberto Pellecchia, Rosa Crestani, Tom Decroo, Rafael Van den Bergh, Yasmine Al-Kourdi

**Affiliations:** 1 Liberia mission, Médecins Sans Frontières–Operational Centre Brussels, Monrovia, Liberia; 2 Operational Department, Médecins Sans Frontières–Operational Centre Brussels, Brussels, Belgium; 3 Medical Department, Médecins Sans Frontières–Operational Centre Brussels, Brussels, Belgium; IFIMAR, UNMdP-CONICET, ARGENTINA

## Abstract

**Introduction:**

In the Ebola Virus Disease (EVD) outbreak in Liberia, two major emergency disease-control measures were cremation of bodies and enforcement of quarantine for asymptomatic individuals suspected of being in contact with a positive case. Enforced by State-related actors, these were promoted as the only method to curtail transmissions as soon as possible. However, as with other harsh measures witnessed by Liberian citizens, in many cases those measures elicited uncontrolled negative reactions within the communities (stigma; fear) that produced, in some cases, the opposite effect of that intended.

**Methodology:**

The research has been conducted in two phases, for a total of 8 weeks. Ethnography of local practices was carried out in 7 neighbourhoods in Monrovia and 5 villages in Grand Cape Mount County in Liberia. 45 Focus Group Discussions (432 participants) and 30 semi-structured interviews sustained the observing participation. Randomly selected people from different social layers were targeted. The principal investigator worked with the help of two local assistants. Perceptions and practices were both analysed.

**Results:**

Participants stressed how cremation perpetuated the social breakdown that started with the isolation for the sickness. Socio-economical divides were created by inequitable management of the dead: those who could bribe the burial teams obtained a burial in a private cemetery or the use of Funeral Homes. Conversely, those in economic disadvantage were forced to send their dead for cremation. State-enforced quarantine, with a mandatory prohibition of movement, raised condemnation, strengthened stigmatization and created serious socio-economic distress. Food was distributed intermittently and some houses shared latrines with non-quarantined neighbours. Escapes were also recorded. Study participants narrated how they adopted local measures of containment, through local task forces and socially-rooted control of outsiders. They also stressed how information that was not spread built up rumours and suspicion.

**Conclusions:**

Populations experiencing an epidemic feel a high degree of social insecurity, in addition to the health hazards. Vertical and coercive measures increase mistrust and fear, producing a counter-productive effect in the containment of the epidemic. On the other hand, local communities show a will to be engaged and a high degree of flexibility in participating to the epidemic response. Efforts in the direction of awareness and community involvement could prove to be better strategy to control the epidemic and root the response on social participation.

## Introduction

The recent Ebola epidemic hit three countries in West Africa (Liberia, Sierra Leone and Guinea) the most, while it was swiftly secured in other countries where it took hold. By the end of May 2015, the disease resulted in 11,162 deaths, on 27,781 cases globally declared [1]. The containment of the largest outbreak of Ebola in human history strained international and local response capacity, distressing local populations, caught in the grip of a disastrous epidemic and a limited public health service. Emergency coercive measures were adopted in Liberia in August 2014, when the epidemic was finally officially declared. Mainly enforced by State-related actors, in Liberia the most significant measures were cremation of bodies and quarantine of contacts (hereinafter, State-imposed quarantine). The objectives of these measures were to quickly interrupt the transmission caused by funerals and by contacts of symptomatic persons. Lifestyles, traditions, and an ill-defined concept of culture were held to be the main responsible of the circulation of the virus by the implementing actors [2–6]. This rationale, as well as the way the measures were imposed, and the communication messages sustaining the emergency actions, created social consequences for the Liberian population.

A body of literature exists on how public health measures negatively impact people without offering a substantial reduction of virus transmission [7–10]. Existing research focuses on theories of public health, their relations with ethics and human rights, and the practicability of measures in different settings and for different infectious diseases. Amongst these, Influenza, Severe Acute Respiratory Syndrome (SARS) and few about Ebola, are the most recent studied, although the effects of quarantine for example have been an issue dating back to the plague [11]. Conversely, little consideration has instead been dedicated to a full analysis of cremation and to communities' direct experiences of coercive measures in public health. How do people perceive interventions to protect their health? What dynamics does coercion trigger? What social practices do communities activate to resist, or respond to, such interventions? To which extent are these measures effective in the light of the social consequences they generate? To address these questions, this study was conducted to assess Liberian community perspectives on State-imposed Ebola public health and outbreak containment measures implemented in 2014 and 2015.

## Methods

### Study design

The research consisted of a qualitative approach based on ethnography of social practices and perceptions.

### Study sites

The research took place in Montserrado (Greater Monrovia and St. Paul Bridge Districts) and Grand Cape Mount (Tewor and Commonwealth Districts) counties. Specifically, the zones in Montserrado were New Kru Town, Congo Town, Paynesville, Clara Town, West Point, and Central Monrovia; and in Grand Cape Mount: Bangoma, Tiene, Diah, Camp 3, and Augustine Village. The areas were purposively selected, on the basis of different criteria: epidemiological incidence of Ebola (cases per neighbourhood or village); presence of quarantined houses; and particular sociological or demographic features (i.e. large religious community; presence of market; bus stations; health facilities). The ethnic composition of Montserrado County is mixed, while the included areas in Grand Cape Mount County are mainly Vai, Mende, and to a limited extent Fula. The governance of the areas is structured around the co-existence of two forms of power, with on the one hand a Government-based political power, with superintendents, commissioners, and chairpersons, all affiliated to a greater or lesser extent to the political parties, and on the other hand the “traditional” system of Chieftaincy. The research dealt with both types of leaders, with an emphasis on those who were based locally (i.e. chairpersons and chiefs) and who were most involved in the daily activities of their communities. Other forms of administration of power and socio-political legitimacy included the “secret societies” of which Poro and Sande are the most important.

### Study period

The fieldwork was carried out in two phases: the first, in October and November 2014, focused on an analysis of the general social response toward the epidemic and in particular the modification of funeral and burial practices. An in-depth enquiry of the perceptions of cremation was conducted during this phase. Data on quarantine was collected in the second phase in January and February 2015.

### Participants

Participants were purposively recruited on a voluntary basis, after full explanation of the goals of the research. All demographic strata of society were included, both males and females, with the exclusion of children. Specific attempts were made to cluster people into groups particularly affected by the epidemic, e.g. public transportation workers; market sellers; and healthcare workers. Both political (government and traditional) and religious (Christian, Muslim, and traditional) leaders were involved as participants. Representatives of the two main secret societies in Liberia (Poro and Sande) were involved both as informants on the practices of their societies, and as opinion leaders in general.

### Data collection

The Principal Investigator worked with two local assistants, trained as data collectors and interpreters. Both assistants held university degrees and were selected on the basis of their background in research, their knowledge of the area and their local language competencies (especially Vai and Mende). One of the two assistants was originally from Grand Cape Mount County. The researchers collected both social practices and narratives (opinions; perceptions; feeling; comments). The first were acquired through participant observation: the researchers visited, and participated in, social activities; gatherings; family reunions; meetings of local leaders; and religious and other daily activities. Reflexivity on the positioning of the researchers into the research settings was permanently performed in order to contain misrepresenting dynamics possibly activated in the relationship between researcher and observed / respondent. Narratives were collected through 45 Focus Group Discussions (FGDs) for a total of 432 participants and 30 semi-structured individual interviews. Narratives were collected in Liberian English, and were transcribed and translated in Standard English if necessary. Translations were checked for consistency by a third Liberian native not involved in the research. Question grids with starter questions were provided to the moderator of FGDs and interviewers, in order to frame the discussion. The grids were organized according to three main themes: 1) general social perception of the epidemic and community's reactions; 2) funerary and burial practices before and during the epidemic, and opinions on cremation; and 3) health-seeking behaviours and perception of quarantine. Each grid contained basic questions but was open to probes and contextual questions. Social status, religious belonging, degree of involvement in the epidemic (e.g. family link with the sick or dead) were considered as patterns of discussion. Narratives were linked with observation of practices and reciprocally used to frame new questions and targets of observation until saturation of information was reached. The following table (Table 1) shows the characteristics of the study participants.

**Table 1:** Characteristics of study participants.

	Participants in FGDs (n)				Participants in interviews (n)			
	Montserrado County		Grand Cape Mount County		Montserrado County		Grand Cape Mount County	
	Male	Female	Male	Female	Male	Female	Male	Female
Political leaders (governmental; traditional; community leaders)	30	13	5	-	1	4	2	-
Religious leaders (christian; muslim; local religions)	35	5	11	-	4	1	2	-
Youth / students	20	15	8	4	-	1	-	-
Market sellers	7	41	-	18	-	2	-	1
Transport workers	48	-	10	-	2	-	2	-
Health workers	4	17	-	-	1	1	1	1
Farmers / miners / fishermen	-	-	15	13	-	-	2	-
Employees / teachers	46	15	5	6	1	1	-	-
Unemployed	8	13	10	10	-	-	-	-
TOTALS	198	119	64	51	9	10	9	2

FGD: Focus Group Discussion; n: number.

### Analysis

Data were analysed through a medical and social anthropological approach. Narratives and observations were recorded and transcribed. Data were compared through triangulation of methodological tools (observation / FGD / interview); time (e.g. during quarantine / after quarantine); space (different neighbourhood or villages); persons (e.g. leader / common person). Themes rising from the interviews and FGDs were compared, taking into consideration social status of the interviewed; kind of involvement in the epidemic (e.g. history of illness; experience of quarantined; experience of death of a relative); area and general social environment where the interviewed acted; the narrative logic of the themes emerging from his/her interview. No software was used: data analysis was carried out through assessment of transcriptions, with key-words and key-concept comparison. Quotes excerpted and used as references in the following article are examples of general understanding.

### Limitations

The research was conducted in a high risk environment, with a mandatory no-touch policy and obvious restrictions. Participant observation was therefore organized without a full involvement of the researchers in the social practices, as common in an anthropological fieldwork, relying on observations and collection of narratives.

### Ethics

Informed consent was obtained verbally following explanation of the purpose of the research, the anonymity of the data gathered, and the voluntary basis of involvement. Research was conducted on the basis of the Principles of Professional Responsibility as declared in the Statement of Ethics of the American Anthropological Association [12]. The full study was approved by University of Liberia-Pacific Institute for Research & Evaluation Institutional Review Board (Monrovia, Liberia).

## Results

### Reactions to State-imposed quarantine

Forced quarantine of asymptomatic contacts of positive cases was the main State-imposed measure that transformed social perceptions and practices. In August 2014 the Government of Liberia commanded the quarantine of an entire neighbourhood in Monrovia, called West Point, considered a slum [13]. West Point was quarantined through the intervention of military forces to respond to the protest of the residents. Many people were injured, and one person died during the clashes. Some students living in the area recalled that day in a FGD:

«*A*: *“*We didn't understand the logic… Instead of helping us with clinics and ambulances they pushed us in. What were we supposed to do? Keep the dead inside? No-one came to educate us…what is Ebola? They talked about a virus that came all in a sudden and the day after we witnessed the police with sticks and guns…”

*B*: “Yes! I saw with my eyes a woman running away from a soldier that wanted to beat her! She run and run and got safe in the church.”

*A*: “That was too bad! And even we knew that they were bringing bodies in West Point from different areas in town. And…why that? I think they wanted to exterminate us”». (men, 21 and 22 years old, West Point, Monrovia, 28/1/2015, S2)

West Point's quarantine paved the way for forced isolation of communities and individuals in Liberia [14]. All participants in the study had a relative, a friend or an acquaintance isolated. State-enforced quarantine has been enforced in several areas of Monrovia and villages in rural counties. While the intensity of the enforcement differed, overall it manifested common characteristics. Movements of the quarantined were restricted for the incubation period (21 days); a daily control of the temperature was planned; distribution of food and items were planned. The research records that few awareness messages were spread to explain the public health measure. The Government imposed the measure through a formal mandate to commissioners, chairpersons, and chiefs of rural villages. However, the local leaders who participated in the research reported a lack of involvement in the decisional forums and felt the form of the State-enforced quarantine did not comply with local communities’ dynamics. In addition, spiritual leaders were not consulted, despite their strong influence in the communities. State-enforced quarantine broke social networks of solidarity and was implemented with lots of gaps that raised social concern. Study participants, both those under quarantine and neighbours, explained how food, water and other items were only intermittently distributed by the implementing agencies, creating harm and forcing residents to disobey the imposed isolation. In Augustine Village, in Grand Cape Mount County, dwellers helped with food and water a woman under quarantine who for days was not receiving assistance. One of the man clearly stated that such help was a common habit among the residents of the small village, and that quarantine threatened this spirit (man, 38 years old, Augustine Village, 15/1/2015, S7). A second consequence was that State-enforced quarantine increased level of stigmatization, which instead was much lower where community managed contacts follow-up. Quarantine triggered a process of publicly labelling those under forced isolation, which created panic, fear and disenfranchisement of minority groups. In Zinc Camp, St. Paul Bridge, Monrovia, communities experienced several houses under quarantine connected to two positive cases. A local religious leader, during a FGD, remarked bitterly how the quarantine brought suspicion and mistrust to his community: he feared that the stigma associated with forced isolation would eventually undermine the mutual trust, forcing those who need help not to ask for it (pastor, 50 years old, St. Paul Bridge, Monrovia, 16/2/2015, S8). Stigma was reported frequently: people isolated were accused of being infected and judged for their alleged controversial social behaviours. The common labelling was “Ebola People”, despite the fact that they were not even infectious. Indeed, quarantine fed rumours and gossip: families that happened to be under quarantine were said to belong to a different ethnic group or religion, and therefore dangerous for being alien. In Tiene, a village in Grand Cape Mount County, a young man conferred the reason of forced isolation of his neighbours on their belonging to the “Fula” tribe. The village hosts a small Fula community, mainly engaged in business activities and vehicle repair. Despite being perfectly integrated and actually Liberian citizens, a subtle popular belief holds that Fula are nomads and therefore foreigners. Due to this, people interviewed in Tiene linked the spread of Ebola–known to come from outside the country–to the Fula. A third consequence of coercive quarantine can be identified in the obstacles to health seeking behaviour. Being blocked into houses, and with the fear of being infectious, people under quarantine kept easily treatable non-Ebola illnesses as a secret. This fostered “informal” medicine providers–such as drugs sellers–who were more mobile than “formal” medicine. The temperature control planned as a way to monitor quarantined, was actually very discontinuous, rooting a perception of a measure not beneficial and purely coercive.

The State-enforced quarantine was imposed vertically, and overshadowed local isolation measures that were already organized by local leaders (such as chairpersons, village chiefs, and opinion leaders) and were more socially accepted. These local measures started as spontaneous and self-organized form of protection, but institutional levels paid very little attention to them, choosing the imposition of power. Indeed, communities did understand that isolation was crucial in case of symptoms. Local leaders, such as chairpersons, organized the limitation of movements in the communities for contacts, inviting them to be reachable, or for foreigners to be identifiable as soon they arrived in the neighbourhood. In case of isolation of contacts, leaders and community-based task forces provided food and water. In most cases, locally developed isolation of asymptomatic contacts was not total. The case of an area near Mount Barclay, Monrovia, illustrates the differences between coercive quarantine and local management of contact follow-up. Two houses with a total of 52 individuals, contacts of two positive cases, were requested to be available for contact tracing by the local chairperson, who later worked in close collaboration with the local contact tracer, visiting the contacts every day. Food and water were provided by the community, but only minimally. In fact, no restriction of movement was imposed, since contacts agreed to be available daily. Here through the words of the local leader and one of the individual contacts:

«I don't use the word quarantine, it is too harsh. I prefer surveillance or monitoring…I can't understand why everyone's using quarantine! When Mr. B. [the positive case] died, all community participated to the mourning, he was part of us. We don't see his relatives as our enemies, they're our fellows. Quarantine cannot work here, it drowned people in fear and economic hardship. We don't ban movements, they are free to go and get food…how they can do otherwise?» (Leader, Mount Barclay, 6/1/2015, S6)

«I am a machinist at R. Factory there. I am here today, but the rest of the people [under isolation] are outside to get food or work […] We received this morning the visit of the contact tracer, she took our temperature and it was normal […] I don't like to stay here all day, but I understand the need–we have to save lives. The chairlady spoke with my boss and he agreed to give me leaves for this period but my brothers are not so fortunate and they have to continue working» (man, 42 years old, Mount Barclay, 6/1/2015, S6)

### Resistance to cremation

Cremation of bodies was another measure taken to curtail transmission. Participants acknowledged that during the months of August and September, the amount of bodies was so high that such a measure was needed. However, they criticized the way it was implemented and the duration of enforcement, continuing cremation even after the number of fatalities decreased, when safe and dignified burials were considered feasible again. Health care workers explained that the rationale of cremation stands on the belief that funerals are locally dangerous and people handle bodies unsafely. However, the narratives of participants do show a high degree of flexibility within communities in changing practices. A significant decrease in unsafe handling of funerals–the so-called “traditional” funerals–was registered and recognized by all respondents, including the representatives of Poro and Sande secret societies, that were believed to be resistant to change. In early October, the leader of one of the biggest Muslim communities in Monrovia published an article in a local newspaper encouraging his fellows to handle deaths in a proper way to avoid the spread of Ebola [15]. This contrasted with a general narrative in the media that claimed resistance among Muslims to changing their practices. Indeed, initial resistance was transversal amongst the different religions, and participant observation in Greater Monrovia showed how some pastors of charismatic churches preached the non-existence of the virus.

The mandatory cremation and the disorganized body collection created an *informal economy of dead bodies*, uncontrolled by Health Authorities and international NGOs. Demands for proper funerals resulted in burials organized by non-official teams that, upon payment of a sum of money, took care of the removal of the body, finding a graveyard and burying the body. However, such a “service” parallel to the official one was not affordable to every family. Research participants reported a sort of “price-list” that these teams presented each time: those who could afford it, paid for the whole process, while the poor where forced either to keep the body at home or to wait for the Burial Team and send the body to the Crematorium. Some participants even claimed that bribery and corruption was normal, and rich families could pay the official burial team to “close an eye” and let them contact a Funeral Home. These dynamics and social behaviours have been labelled by the media as “secret burials” although they were very well known by locals in Monrovia. Secret burials” was used especially by the media and NGOs as a catchphrase to identify very different practices. These practices included the “secret burials” allegedly performed by the secret societies Sande and Poro and “secret” burials” which resulted as a form of resistance to the mandatory cremation,. The latter had little to do with the secret societies, and were “secret” in the meaning of being hidden to institutional Government-based authorities. While they were performed “in secret”, their existence was hardly hidden, and circulating around the city it was possible to observe burials performed in the small private cemeteries spread throughout.

All study participants confirmed through empirical examples the following situations that frequently occurred in Monrovia until the cemetery started its activities:

- families calling the national hotline of the Ebola Task Force to activate the burial teams for picking up a body and not receiving any answer;
- families waiting for burial teams for hours or even days, with dead bodies in the streets or indoors;
- long waits for the non-Ebola certification in order to bury the non-Ebola bodies and consequent development of an informal market of certificates, quite expensive for the average local income;
- several cases of non-Ebola deceased sent to cremation because of fear;
- in the event of death, families were hesitant to involve the Ebola Task Force to remove the body since they knew that it would be sent to the Crematorium. The body was therefore kept at home and secretly buried in private cemeteries in town or sent in the villages of origins in the countryside;
- upper classes of Monrovia's society could afford the involvement of Funeral Homes to quickly obtain certificates of non-positivity to EVD and therefore organize a funeral and burial. Before the epidemic, Funeral Homes were normally used by all families despite their economic condition. During the epidemic such firms increased the prices of the different services operating a consequent, although indirect, selection of the customers.

The following quote from a FGD held in Paynesville, Monrovia, exemplifies the communities' feeling around the situations listed:

«When I saw that body on the road I felt very bad. It stayed there for hours under the sun! And nobody came to pick up…we called, called, called. The chairman too he called the MOH but nobody picked. And then–I tell you!–I understand those that keep the body in, which is unsafe, but at least they can do a proper funeral and cry for it. I'd do the same: just pay someone to dig the grave and you know where your beloved is.» (man, 40 years old, Red Light, Monrovia, 26/10/2014, S3)

### General social perception on outbreak control

Participants in FGDs and interviews agreed that outbreak response managed by State-related actors created a climate of fear that did not help them to understand the causes, ways of transmission, and prevention strategies. They stressed how the awareness campaign relying on harsh messages–such as “Ebola is a killer!”, “Ebola can kill anyone!” or “Last warning: Ebola is real!”–frightened them to the point that they believed contagion was unavoidable. This feeling guided their health behaviours, as the following quote evokes:

«If they say 'Ebola Kills! Ebola Kills!', then what you should do? I prefer to die at home, if I have to die anyway.» (woman, 49 years old, Congo Town, Monrovia, 13/10/2014, S5)

Although messages evolved to a less drastic tone throughout the outbreak, fear and insecurity continued to shape people's perceptions. The outbreak was described as exploiting social bonds and creating mutual distrust. Containment measures duplicated such feelings and revived the time of civil war, with its climate of insecurity and suspicion towards neighbours, close acquaintances and foreigners. As during the war, all but the family were perceived as a potential enemy. A participant to a FGD pictures his experience as such:

«Now you can't trust anyone–not your mother, not your father, not your wife. Everyone's a danger. Your dead too are a danger […] This is a breakdown, a breakdown of our habits, of our traditions. We will never return back.» (man, 34 years old, Elwa, Monrovia, 9/10/2014, S1).

During an interview, a local leader said: «Ebola first kills and then steals» (man, 49 years old, New Kru Town, Monrovia,12/10/2014, S4), to describe the *modus operandi* of cremation that “steals” the beloved.

## Discussion

Cremation of bodies and forced quarantine, as ways to quickly reduce transmission of Ebola outbreak for the benefit of the larger public, produced social dynamics of resistance in the same population that they wished to protect. On one hand, nonconformity to cremation was based on the socio-cultural and economic importance conferred by Liberian society to funerals. Unequal and drastic imposition of cremation contributed to condemnation of a measure that, at least initially, was understood as a containment strategy. On the other hand, forced quarantine of asymptomatic contacts tore up an already broken social material, fuelling fears and mutual mistrust that did not help promotion of health seeking behaviours. Respondents attributed the main reason of the social resistance to coercive measures to the experience of war that fragmented the society as well as the epidemic [16].

Scholars in law, public health, and bioethics show how enforcement of coercive measures in communicable diseases may possibly help contain an outbreak, but since they operate through command, they result in a huge social harm, which threatens the same outbreak control strategy [10, 17–19]. As the observations and interviews of this research reveal, similar observations can be seen in the case of Ebola in Liberia, where coercive “public” health measures produced the opposite effect of distancing individuals (the “public”) to implementing agencies [20–21]. An anthropological perspective allowed an understanding of socio-political implications of public health measures, analysing how these are practically experienced and performed by people [22–25]. The set of dynamics that sprang from Liberian society while facing the epidemic appeared to be coherent responses to imposed devices not perceived as helpful. The quarantine increased the level of insecurity and fear, forcing people under isolation to avoid seeking care, and neighbours to stigmatize and deny.

The social response of Liberians to State-enforced, vertical, quarantine, as described by participants, demonstrated a thin boundary between collective responsibility and respect of individual rights and civil liberties. The argument of interrupting transmission through vertical measures was not balanced by a guarantee of basics for living, social protection and individual freedom. Although *top-down* quarantine has shown its public health limits in several past occasions [17], and even though it has been strictly regulated in USA and Europe, it was indiscriminately used in Liberia [19, 26], with means inconceivable in the Western World, such as military force [27]. On the contrary, less invasive forms of isolation can be developed at community level, and can be practiced with the consensus of involved citizens, supported by close leaders and a network of acquaintances. Cremation was for the first time widely performed as an outbreak control measure in Liberia. Despite a “technical” success in quickly eliminating bodies, its unintended consequence was the emergence of burials performed in secret, often with unsafe practices, indirectly promoting what can be termed an *informal economy of death*. Then, sustained by the idea that traditions were wrong and funerals were the main cause, implementation of mandatory cremation crudely defined the transition from life to death as a simple biomedical passage of state, wiping out deep social links, and endangering the credibility of the measure itself.

Indeed, both measures seemed to underscore that the local population's health should be ruled instead of managed. Three major implications emerge from this research. First, as stressed by the narratives of the informants, alienating the participation of populations and excluding their involvement, leads to non-compliance and increases risk of virus transmission. A community-based isolation of contacts, managed locally by leaders, health authorities, and community-based organizations, would likely have been a more acceptable alternative to vertical quarantine. Second, a *top-down* militarized-like measure does not allow the population to understand the emergency situation, creating a distance between institutions and citizens [14]. Third, coercion does not directly result in a change of practices or benefit health-seeking behaviours. On the contrary, enforcement of vertical measures is seen as a command, an injustice and a judgment of allegedly untoward behaviours. Such mechanisms feed self-organization as a form or resistance, but also a creation of informal economies that could be hazardous for public health and increase socio-economic divides. Throughout the epidemic, safe and dignified funerals should been sustained as a clear sign of respect and understanding of the complex socio-cultural and spiritual significance of the dead, even in times of crisis. Cremation, for example, could have been promoted as a temporary measure and organized through the involvement of community leaders–in particular those more in touch with people, such as chairpersons, chiefs, pastors and imams. Family of the deceased could have been involved and could have been returned the ashes. Again, local patterns of funerals could have been identified, discussed with health promoters in collaboration with recognized local leaders, to plan a prevention of transmission within the families [28].

In postcolonial environments such as those mainly affected by Ebola in 2014 and 2015 already characterized by inequalities, fear, insecurity and stigma constitute the social aspect of the disease outbreak. This research supports a comprehensive approach to outbreak control, where health is considered in its totality of shades, from the biomedical to the social. In this sense, an understanding of the drivers of fear and mistrust in the affected communities which ultimately result in behaviour that may increase disease transmission, appear to be a crucial and substantial part of an outbreak control.
